# Cure of Corns

**Published:** 1857-12

**Authors:** 


### CURE OF CORNS.

SOAK the foot in warm water for about a quarter of an hour, every night; after each soaking, rub on the corn patiently, with the finger, half a dozen drops of sweet oil, wear around the toe during the day two thicknesses of buckskin, with a hole in it to receive the corn, continue this treatment until the corn falls out; and by wearing *moderately* loose shoes, it will be months, and even years, before the corn returns, when the same treatment will be efficient in a few days.

Paring corns is always dangerous, besides making them take deeper root—as will a weed, if cut off near the ground. Many applications are recommended to be made to corns, to burn or eat out, or soften them, but the plan advised above is *safe*, is PAINLESS, gives most welcome relief in a few hours, and prevents a return of the corn for a longer time than any other remedy; and last of all, it costs nothing but a little attention: that, however, is the great drawback; if the sweet oil could be disguised, and obtained from some Indian, who expressed it from a rare plant, growing in the caverns of the Rocky Mountains, and sold at a dime a drop, it would have as great a run as the Hemp Juice, which is peddled so successfully by the man whose "Sands of life have been *almost* run out," for some years past, under the name of *Cannabis Indica.* Glycerine, the essential principle of oils, is milder and better than oil itself, as it remains longer moist, and thus has greater softening powers.

